# Clinicopathological features and treatment outcomes of the rare, salivary duct carcinoma of parotid gland

**DOI:** 10.1186/s40463-016-0146-2

**Published:** 2016-05-16

**Authors:** Khalid Hussain AL-Qahtani, Mutahir A. Tunio, Yasser Bayoumi, Venkada Manickam Gurusamy, Fahad Ahmed A. Bahamdain, Hanadi Fatani

**Affiliations:** Department of Otolaryngology-Head & Neck Surgery, College of Medicine, King Saud University, Riyadh, Saudi Arabia; Radiation Oncology, NCI, Cairo University, Cairo, Egypt; Radiation Oncology, King Fahad Medical City, Riyadh, 59046 Saudi Arabia; Faculty of Medicine, King AbdulAziz University, Riyadh, 59046 Saudi Arabia; Histopathology, Comprehensive Cancer Center, King Fahad Medical City, Riyadh, 59046 Saudi Arabia

**Keywords:** Salivary ductal carcinoma, Parotid gland, Saudi population

## Abstract

**Background:**

Salivary ductal carcinoma (SDC) of parotid gland is a rare and aggressive entity; accounting for 1–3 % of all malignant salivary gland tumors, 0.2 % of epithelial salivary gland neoplasms, 0.5 % of salivary gland carcinomas, and 1.1 % of parotid gland carcinomas. Here in we aimed to evaluate the clinico-pathological features and treatment outcomes of parotid gland SDC in Saudi population.

**Methods:**

Among 38 patients with parotid malignancies, who were treated in two major tertiary care referral cancer centers between December 2007 and December 2014, seven cases (18.4 %) were found to have SDC, which were investigated for clinicopathological features, locoregional recurrences (LRRs), distant metastasis (DM) and survival rates.

**Results:**

Mean age of cohort was 62.3 years (range: 41–83) and female predominant (71.4 %). All patients underwent total parotidectomy and ipsilateral neck dissection. Mean tumor size was 3.4 cm (range: 2.1–5.3); perineural invasion (85.8 %); lymph node involvement (42.9 %); and HER-2 neu overexpression (28.6 %). Postoperative radiation therapy (PORT) was given to six patients (dose: 50–66 Gy). Median follow-up was 20.2 months (range: 11–48). LRRs were seen in five (71.4 %) patients (base of skull, 3 patients; cervical nodes, one patient; parotid bed, one patient). LRRs were salvaged with resection (two patients) and re-irradiation (one patient with base of skull). DM in lungs was seen in three patients (42.8 %); one treated with carboplatin/paclitaxel based chemotherapy. The 4-year disease free and overall survival rates were 16.7 % and 40 % respectively.

**Conclusion:**

SDC of parotid gland is a rare and aggressive entity, and most of LRRs were seen in the base of skull, which warrants inclusion of base of skull in clinical target volume in PORT planning. Role of anti HER-2 targeted therapy in SDC with HER-2 neu overexpression needs further investigations.

## Background

Salivary ductal carcinoma (SDC) of parotid gland is a rare and aggressive entity; accounting for 1–3 % of all malignant salivary gland tumors, 0.2 % of epithelial salivary gland neoplasms, 0.5 % of salivary gland carcinomas, and 1.1 % of parotid gland carcinomas [1, 2]. SDC of parotid gland has been classified as high grade tumors along with high-grade mucoepidermoid carcinoma and carcinoma ex pleomorphic adenoma in the updated World Health Organisation (WHO) classification of salivary gland tumors [3]. The histopathological features of SDC of parotid gland are similar to those of breast ductal carcinoma requiring a differential diagnosis with possible metastasis through immunohistochemistry (IHC) analysis among patients with a previous history of breast carcinoma [4].

The standard treatment for SDC of parotid gland is total parotidectomy, ipsilateral neck dissection followed by postoperative radiation therapy with or without concurrent chemotherapy; however, SDC of parotid gland has grave dismal prognosis [5].

Here in, we describe and discuss the clinicopathological characteristics and treatment outcomes SDC of parotid gland in our population.

## Methods

After formal approval from the institutional review committee, medical records of 38 patients with confirmed parotid gland malignancies, who were treated in two cancer centers of Riyadh, Saudi Arabia during the period of December 2007 and December 2014, were reviewed using digital database system. Patients with SDC of parotid gland were retrieved in following manner;

### Demographic, clinicopathological and radiological variables

Demographic and clinical data including age at the diagnosis, gender, and signs and symptoms at the time of presentation were reviewed. A detailed second review of all histopathological specimens was performed by experienced histopathologist. Different histopathological parameters, including the tumor size, lymphovascular space invasion (LVSI), perineural invasion (PNI), margin status, lymph node involvement and tumor, lymph node and metastasis (TNM) staging were recorded. Data from different imaging modalities, including computed tomography (CT) scan of neck and chest, magnetic resonance imaging (MRI) and flourodeoxyglucose positron emission tomography (FDG-PET) was collected. Data regarding different treatment modalities, including the type of parotidectomy and neck dissection, postoperative radiation therapy (PORT), and its doses were also recorded.

### Statistical analysis

The primary endpoint was locoregional control (LRC). Secondary points were the distant metastasis control (DMC), disease free survival (DFS) and overall survival (OS) rates. Locoregional recurrence (LRR) was defined as, the duration between the parotidectomy and the date of clinically or radiologically detectable disease in the parotid bed or in cervical lymph nodes on imaging. Distant metastasis (DM) was defined as, the duration between the parotidectomy and the date of documented disease outside the neck on imaging. Similarly, DFS was defined as, the duration between the parotidectomy and the date of documented disease reappearance/relapse, death from cancer and/or last follow-up (censored). The OS was defined as, the duration between the surgery and the date of patient death or last follow-up (censored). Probabilities of LRC, DMC, DFS and OS rates were shown with the Kaplan-Meier method, and the comparisons for various survival curves were performed using log rank. All statistical analyses were performed using the computer program SPSS version 16.0. Relevant literature was searched through PubMed/MEDLINE, CANCERLIT, EMBASE, Cochrane Library database, Web of Science, Academic Search Premier, and CINAHL using the terms “(salivary duct carcinoma, ductal carcinoma parotid, Stensen duct carcinoma of parotid. These terms were then combined for search for prospective, retrospective, randomized, controlled, review articles.

## Results

Among thirty eight patients with parotid malignant tumors who were treated in our centers between December 2007 and December 2014, seven cases (18.4 %) were found to have SDC. Mean age of cohort was 62.3 years (range: 41–83), with female preponderance (71.4 %). The common presentation at the diagnosis was the parotid swelling. In two patients (28.6 %), facial nerve palsy was seen at the time of diagnosis. Patient characteristics are shown in Table 1.

**Table 1:** Patient characteristics

Patient	Age /gender	Symptoms	Treatment	Pathology	Recurrence	Metastasis	Died	Follow-up period
1.	52/F	Left parotid swelling, facial nerve palsy	Total parotidectomy and ipsilateral MND	Tumor size: 2 × 3 cm; LVSI -: PNI-; 0/20 LN; HER-2 neu +++; Margins-;	Base of skull with ICE (VI,VIII,IX,X,XI CN palsy) Treated with Palliative RT 25 /10	Bilateral Lungs	Yes	14 months
2.	41/M	Right parotid swelling	Total parotidectomy + ipsilateral MND → RT 60 Gy/30 fractions	Tumor size: 3 × 3 cm,; LVSI-, PNI+; 0/30LN; HER-2 neu +++; margins-	No	No	No	11 months
3.	83/M	Right parotid swelling	Total Parotidectomy + ipsilateral MND → RT 50 Gy/25 fractions	Tumor size: 2 × 2 cm,; LVSI-;PNI+; 0/10 LN; HER-2 neu -; margins+,	No	Bilateral Lungs	Yes	13 months
4.	43/F	Right parotid swelling	Total parotidectomy + ipsilateral MND → 66 GY/33 fractions	Tumor size: 4 × 3 cm,; LVSI+; PNI+; HER-2 neu -; skin +; 2/30LN+; margins+	Mastoid air cells and base of skull Treated with Reirradiation 60 Gy/30 fractions	No	No	27 months
5.	65/F	Right parotid swelling	Total parotidectomy + ipsilateral MND → RT 66 Gy/33 fractions	Tumor size: 4 × 4 cm; LVSI-; PNI+; HER-2 neu -; 5/14 LN+; margins+,	Ipsilateral Neck nodes level III, IV Treated with salvage LND	No	No	12 months
6.	81/F	Right parotid swelling, facial nerve palsy	Total parotidectomy + ipsilateral MND → RT 66 Gy/33 fractions	Tumor size: 5 × 5 cm; LVSI+; PNI+; HER-2 neu -; 3/21LN+; margins +	Mastoid air cells and base of skull treated with chemotherapy	Bilateral Lung treated with Carboplatin/Paclitaxel chemotherapy	Yes	16 months
7.	71/F	Left parotid swelling	Total parotidectomy + ipsilateral MND → RT 60 Gy/30 fractions	Tumor size: 3 × 3 cm; LVSI +; 0/22LN; PNI+; HER-2 neu -; margins-	Tumor bed Treated with resection Pathology 1.2 cm SDC	No	No	48 months

*F* female, *M* male, *MLND* modified neck dissection, *RT* radiation therapy, *LVSI* lymphovascular space invasion, *PNI* perineural invasion, *LN* lymph nodes, *ICE* intracranial extension, *SDC* salivary duct carcinoma

**Fig. 1:**
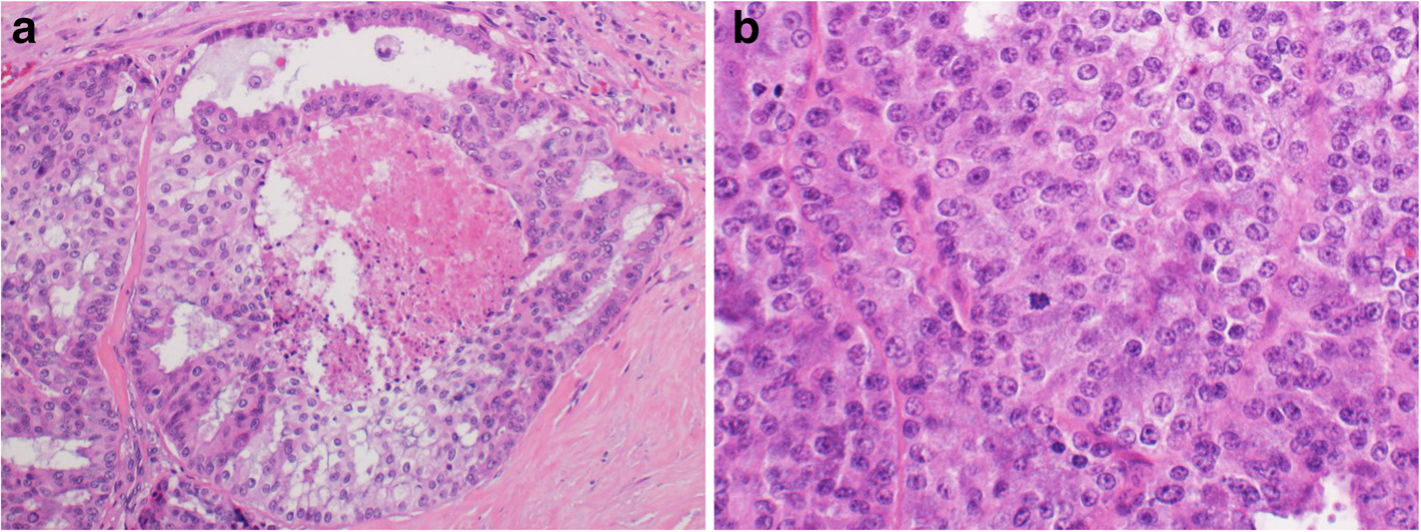
**a** Neoplasm is comprised of cribriform growth pattern with central comedo necrosis (H & E stain, 200 X magnifications); **b** neoplastic cells have polygonal morphology, distinct cell borders, moderately pleomorphic nuclei and increased mitotic activity (H & E stain, 400 X magnifications)

All patients underwent total parotidectomy and modified ipsilateral neck dissection. Mean tumor size was 3.4 cm (range: 2.1–5.3). Predominant histopathological pattern was the neoplasm was comprised of cribriform growth pattern with central comedo necrosis, and tumor cells were polygonal with distinct cell borders, with pleomorphic nuclei and increased mitotic activity (Fig. 1a and b). PNI was observed in 6/7 cases (85.8 %), while LVSI was seen in 3/7 patients (42.9 %). Lymph node involvement was observed in 3/7 cases (42.9 %). HER-2 neu was overexpressed in 2/7 cases (28.6 %).

PORT via IMRT was given to 6/7 cases (85.8 %). Indications were positive margins (66.7 %), and lymph node metastasis (50 %). Median delay between surgery and PORT was 6 weeks (range: 5.5–8). The treatment fields encompassed the tumor bed and upper neck (4 patients), and tumor bed and entire neck (2 patients). Cranial border of PORT fields were kept at base of skull in 2 patients. Mean dose for PORT was 61.3 Gy (range: 50–66 Gy), given as daily 2 Gy/fraction, 5 days/week over 6–6.5 weeks (30–33 fractions).

**Fig. 2:**
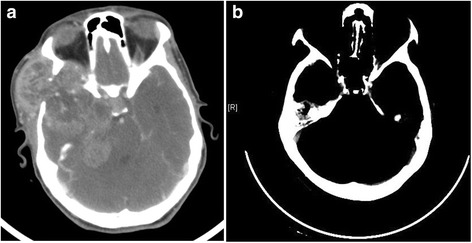
**a** CT head axial image (patient # 1) showing a destructive recurrent mass in the right sphenoid wing, extending into middle and posterior cranial fossae with destruction of a large portion of the skull base; **b** CT head (patient # 4) demonstrating heterogeneous soft tissue recurrent mass involving the right mastoid air cells, mastoid bone and occipital bone associated with bone destruction

**Fig. 3:**
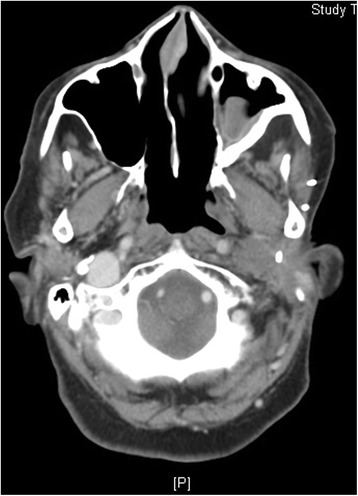
Magnetic resonance imaging (MRI) face showing recurrent mass in parotid bed

**Fig. 4:**
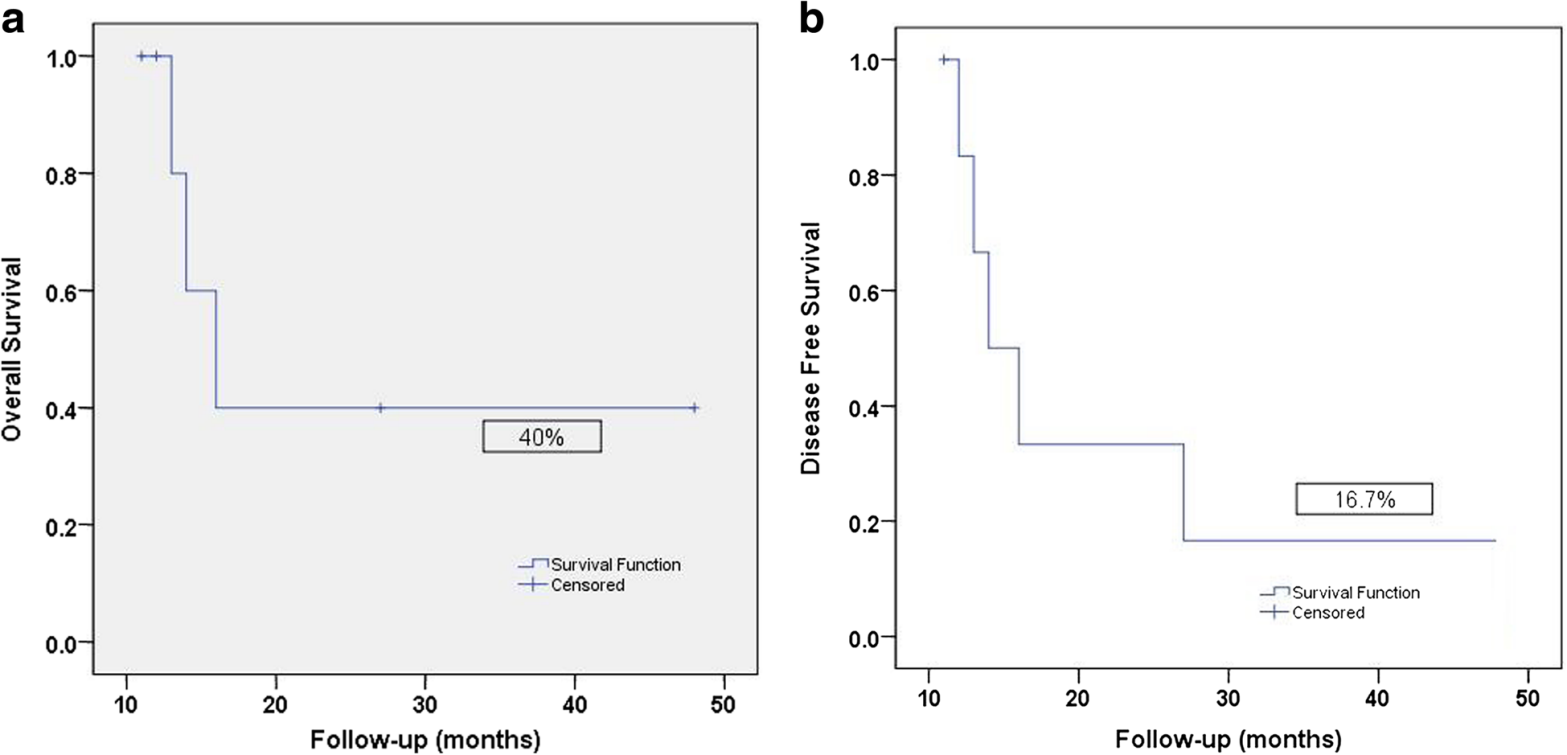
**a** 4- year OS and **b** 4-year DFS rates for SDC in our cohort

Median follow-up was 20.2 months (range: 11–48). LRRs were seen in five (71.4 %) patients. One LRR was in patient without PORT, Two LRRs were marginal near PORT fields (mastoid air cells/base of skull; 3 patients) Fig. 2a and b; two LRRs were seen in-field PORT (cervical nodes; one patient, parotid bed; one patient) Fig. 3. LRRs were salvaged with resection (two patients) and re-irradiation via IMRT (one patient with base of skull recurrence) and systemic chemotherapy (one patient). DM in lungs was seen in three patients (42.8 %); one treated with carboplatin/paclitaxel based chemotherapy. At the time of analysis, four patients (57.2 %) were alive and were disease free. The median time to survival was 15.8 months. The 4-year LRC, DMC, DFS and OS rates were 20.8 %, 40 %, 16.7 % and 40 % respectively Fig. 4a and b.

## Discussion

SDC is an extremely rare and aggressive malignancy of the salivary glands. Owing to its rare nature, clinical data is scanty and only a few clinical studies comprise more than 50 SDC patients [4, 6–8]. To date, largest SDC study has been reported from the Surveillance, Epidemiology, and End Results (SEER) database, based on 228 patients [9]. This review reported that poor prognostic factors for OS and DFS in SDC patients were age 50 years or above, tumor size, and lymph node involvement. However, this study was criticized for the diagnostic bias. Similar other studies have been mentioned in Table 2 [3, 4, 6–8, 10–21]. However, none of these studies has evaluated clinico-pathological features, DFS and OS in SDC of the parotid gland separately, which was the aim of present study. Reason for lower incidence of facial nerve involvement at the time of diagnosis (28.6 %) in our series as compared to reported data can be explained by the fact that the preoperative facial nerve function was not available for many patients, so facial nerve sacrifice at the time of parotidectomy was used as a surrogate for preoperative facial nerve palsy.

**Table 2:** Previously published studies on salivary duct carcinoma of Parotid gland

Study	Patients	Parotid SDC	LN +	PNI	HER-2 neu expression	PORT	Follow-up Mean	LRR	DM	OS
Huang X, et al. [3]	117 M (63.6 %)	7 (63.6 %)	6 (54.5 %)	5 (45.5 %)	9 (81.8)	8 (72.7 %) Dose: 50–60	30 months	2 (18.2 %)	2 (18.2 %)	2-year 75 %
Jaehne M, et al. [4]	50 30 M (60 %)	39 (78 %)	28 (56 %)	-	-	36 (72 %)	24 months	24 (48 %)	24 (48 %)	66 %
Gilbert MR, et al. [6]	75 53 M (71 %)	62 (83 %)	54 (72 %)	52 (69 %)	23 (31 %)	31 (41 %)	55 months	0	0	3 year 50 %
Johnston ML, et al. [7]	54-	49 (90.7 %)	38 (67 %)	-	-	48 (89 %) Dose: 60 Gy	68.4 months	16 (29.6 %)	28 (51.8 %)	43 %
Roh JL, et al [8]	56	34 (62 %)	38 (67 %)	-	-	21 (37.5)	60 months		71 %	44 %
Kim TH, et al. [10]	15 12 M (80 %)	12 (80 %)	9 (60 %)	8 (53.3 %)	-	15 (100 %)	38 months	2 (13.3 %)	7 (47 %)	93 %
Shi S, et al. [11]	38 36 M (94.7 %)	38 (100)	-	-	-	-	48 months	-	-	45 %
Kim JY, et al. [12]	35 30 M (85.7 %)	22 (62.9 %)	26 (74.3 %)	12 (34.3 %)	-	31 (88.6 %)	36 months	9 (25.7 %)	6 (17.2 %)	55.1 %
Brandwein-Gensler M, et al. [13]	19	-	-	-	10 (52.6 %)	-	30 months	-	-	68.4 %
Luna MA, et al. [14]	30 19 M (63.3 %)	24 (80 %)	16 (66.7 %)	-	-	-	24 months	16 (66.7 %)	16 (66.7 %)	30 %
Colmenero Ruiz C, et al. [15]	9 7 M (77.8 %)	8 (88.9 %)	3 (37.5 %)	-	-	7 (87.5 %)	30 months	5 (55.6 %)	3 (33.5 %)	33 %
Delgado R, et al. [16]	15 12 M (80 %)	13 (86.7 %)	2 (13.3 %)	-	-	9 (60 %)	-	5 (33 %)	6 (40 %)	47 %
Guzzo M, et al. [17]	26 14 M (53.8 %)	21 (80.7 %)	15 (57.7 %)	-	-	17 (65.4 %)	60 months	11 (42.3 %)	12 (46.2 %)	46 %
Hosal AS, et al. [18]	15 7 M (46.7 %)	12 (80 %)	11 (73.3 %)	-	-	14 (93.3 %)	48 months	8 (53.3 %)	7 (46.7 %)	43 %
Ko YH, et al. [19]	27 16 M (56.3 %)	21 (77.8 %)	10 (37.1 %)	-	-	19 (70.4 %)	30 months	9 (33.3 %)	15 (55.6 %)	44 %
Afzelius LE, et al. [20]	12 7 M (58.3 %)	12 (100 %)	5 (41.7 %)	-	-	12 (100 %)	60 months	3 (25 %)	6 (50 %)	42 %
Lewis JE, et al. [21]	26 17 M (65.4 %)	23 (88.5 %)	19 (73.1 %)	-	-	13 (50 %)	60 months	15 (57.7 %)	11 (43 %)	43 %
Our study	7 2 M (28.6 %)	7 (100 %)	3 (42.9 %)	6 (85.8 %)	2 (28.6 %)	6 (85.8 %)	20.2 months	5 (71.4 %)	3 (42.8 %)	40 %

*SDC* salivary duct carcinoma, *M* male, *LN* lymph nodes, *PNI* Perineural invasion, *PORT* postoperative radiotherapy, *LRR* locoregional recurrence, *DM* distant metastasis, *OS* overall survival

Our series was predominantly female gender (71.4 %), which is in agreement with one study by Hosal AS et al. [15], while other studies have shown a male preponderance ranging from 53.8 % to 94.7 % [11, 19]. In present study, relatively high incidence of pathological positive cervical lymph nodes (42.9 %) was found in agreement with reported literature, and it warrants routine use of prophylactic ipsilateral neck dissection in SDC of the parotid gland. Similarly, the percentage of pathologically positive PNI (85.8 %) was significantly high in our series, which supports the notion that CTV should include the cranial nerves involved and the corresponding parts of the base of skull in cases of pathologically positive PNI [22]. Interestingly, in our series, no contralateral neck recurrence was seen, therefore the role of prophylactic contralateral neck irradiation needs further investigation.

HER-2 neu overexpression (28.6 %) was much lower than those reported in literature [3, 6, 13]. Recent data has shown that HER-2 neu overexpression and targeted therapy with Trastuzumab therapy is associated with improved DFS and OS rates [23]. Given the limited published data on use of adjuvant or maintenance Trastuzumab in SDC of parotid gland, it might also be useful to develop future Trastuzumab trials in SDC from HER-2 neu positive breast cancer [24]. As SDC of parotid gland has morphologic and molecular similarity to breast cancer, it is recommended that apart from regular histopathological examination, additional immunohistochemical staining including HER-2 neu, Ki-67, p16, p53, estrogen receptors (ER), progesterone receptors (PR), epithelial membrane antigen (EMA), and carcinoembryonic antigen (CEA) should be performed, as proposed by many studies [25, 26].

In contrast to other studies, about 60 % of LRRs were seen in base of skull (PORT was given in 2 patients) in our series, which further supports the hypothesis of inclusion of base of skull in CTV during PORT for these patients as in patients with parotid gland adenoid cystic carcinoma [27]. One LRR in base of skull was salvaged by re-irradiation using IMRT with acceptable toxicity. However, data from re-irradiation in adenoid cystic carcinoma has shown that most of LRRs following re-irradiation occur within the re-irradiated high-dose region, therefore more data regarding dose escalation and delayed toxicity is required [28].

Our study has few limitations. A relatively small number of patients were studied, due to the rarity of SDC in our population. Further, PORT fields, techniques and doses varied somewhat in our study.

## Conclusions

SDC of parotid gland is rare and aggressive entity. Despite extensive treatment by parotidectomy, neck dissection and PORT; a large proportion of patients developed all-sites recurrences. Base of skull should be included routinely in CTV during PORT, and HER-2 neu status should also be examined routinely in all these patients. Further, large multi-institutional studies regarding the role of re-irradiation, systemic chemotherapy, trastuzumab are warranted to suggest optimal treatment approaches for SDC of parotid gland.
